# Interfacial wettability and mass transfer characterizations for gas–liquid–solid triple‐phase catalysis

**DOI:** 10.1002/EXP.20210046

**Published:** 2022-04-16

**Authors:** Run Shi, Lu Shang, Chao Zhou, Yunxuan Zhao, Tierui Zhang

**Affiliations:** ^1^ Key Laboratory of Photochemical Conversion and Optoelectronic Materials Technical Institute of Physics and Chemistry Chinese Academy of Sciences Beijing China; ^2^ Center of Materials Science and Optoelectronics Engineering University of Chinese Academy of Sciences Beijing China

**Keywords:** contact angle, electrocatalysis, finite element, photocatalysis, wetting state

## Abstract

Heterogeneous catalysis is inseparable from interfacial mass transfer and chemical reaction processes determined by the structure and microenvironment. Different from high‐temperature thermochemical processes, photo‐ and electrocatalysis operated at mild conditions often involve both gas and liquid phases, making it important but challenging to characterize the reaction process typically occurring at the gas–liquid–solid interface. Herein, we review the scope, feasibility, and limitation of ten types of currently available technologies used to characterize interfacial wettability and mass transfer properties of various triple‐phase catalytic reactions. The review summarizes techniques from macroscopic contact angle measurement to microscopic environment electron microscopy for investigating the wettability‐controlled structure of triple‐phase interfaces. Experimental and computational methods in revealing the interfacial mass transfer process have also been systematically discussed, followed by a perspective on the opportunities and challenges of advanced characterization methods to help understand the fundamental reaction mechanism of triple‐phase catalysis.

## Graphical abstract

The review focuses on the strategies for evaluating the wettability‐controlled mass transfer process at triple‐phase interfaces. Ten types of characterizations are described based on photocatalytic and electrocatalytic reactions involving water and gas molecules. Finally, the review discusses the tasks of advanced characterizations to help understand the fundamental reaction mechanism for triple‐phase catalysis.

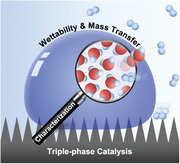

## 1 INTRODUCTION

Energy‐intensive chemical processes such as ammonia synthesis and ethylene production occupy 10–20% of total industrial energy consumption in major global economies, consuming fossil fuel feedstocks and releasing greenhouse gases in a giant quantity.^[^ ^1^, ^2^ ^]^ The green transformation of the chemical industry is an important segment to achieve the goal of carbon neutrality.

As an important branch of interface science, heterogeneous catalysis has been widely studied and applied to the modern chemical industry in the past century.^[^ ^3^ ^]^ One primary goal of heterogeneous catalysis is achieving higher reaction activity at milder conditions. This is a tough challenge from a chemical reaction kinetics viewpoint. Lower reaction temperature indicates a weaker collision between reactants and catalysts, thus leading to less opportunity to overcome the reaction barriers. Thousands of literature have been published each year to understand and optimize the intrinsic reaction kinetics over well‐designed catalytic active sites.^[^ ^4^, ^5^, ^6^ ^]^ Actually, low reaction temperatures and pressures can also result in poor reactant diffusivity and concentration, leading to an insufficient supply of reactants to the surface of catalysts.^[^ ^7^, ^8^ ^]^ This limitation in physical mass transfer kinetics may cause limited molecule collisions, further hindering the catalytic activity enhancement at mild conditions. In addition, different from various gas–solid double‐phase reaction systems in thermocatalytic processes (Haber‐Borsch ammonia synthesis, Fischer‐Tropsch synthesis, etc.), most energy and environmental catalysis driven by renewable light or electric energies (such as water splitting, CO_2_ reduction, nitrogen fixation, and aerobic oxidation) needs water as the most important clean proton sources.^[^ ^9^, ^10^, ^11^, ^12^ ^]^ Water exists in the liquid state at low temperatures with poor saturated vapor pressure and shows limited diffusion coefficient and saturated solubility toward gas molecules.^[^ ^13^ ^]^ Therefore, the effective contact between gas/liquid reactants and solid catalysts becomes an essential scientific and engineering task for gas‐consuming and gas‐evolving catalytic reactions.

The contact between gas, liquid, and solid substances as a basic phenomenon in nature has been studied for centuries. Since the first theoretical framework done by Wenzel in the 1930s, physicochemical processes over triple‐phase interfaces have gradually become a research hotspot, including superwettability, one of the top ten emerging technologies in chemistry 2021 established by IUPAC.^[^ ^14^, ^15^ ^]^ In contrast to conventional double‐phase photo‐ and electrocatalytic systems, which consist of catalysts immersed in a bulk liquid phase, triple‐phase catalytic systems by supporting catalysts at gas–liquid boundaries have been developed and exhibited outstanding performance.^[^ ^16^, ^17^, ^18^ ^]^ A couple of reviews have focused on the rational design of triple‐phase catalysts and reactors.^[^ ^19^, ^20^, ^21^, ^22^ ^]^ The enhancement is typically ascribed to the fast gaseous reactants supply and products departure.^[^ ^23^, ^24^ ^]^ Although pioneer works have shown the great opportunity of triple‐phase catalysis in promoting the reaction kinetics at mild conditions, the fundamental reaction process at the interface has yet to be well understood.^[^ ^25^, ^26^ ^]^ The exact answer relies on developing advanced characterization technologies to unveil the wettability of nanostructured triple‐phase interfaces and their influence on the interfacial mass transfer and chemical reaction properties.

**FIGURE 1:**
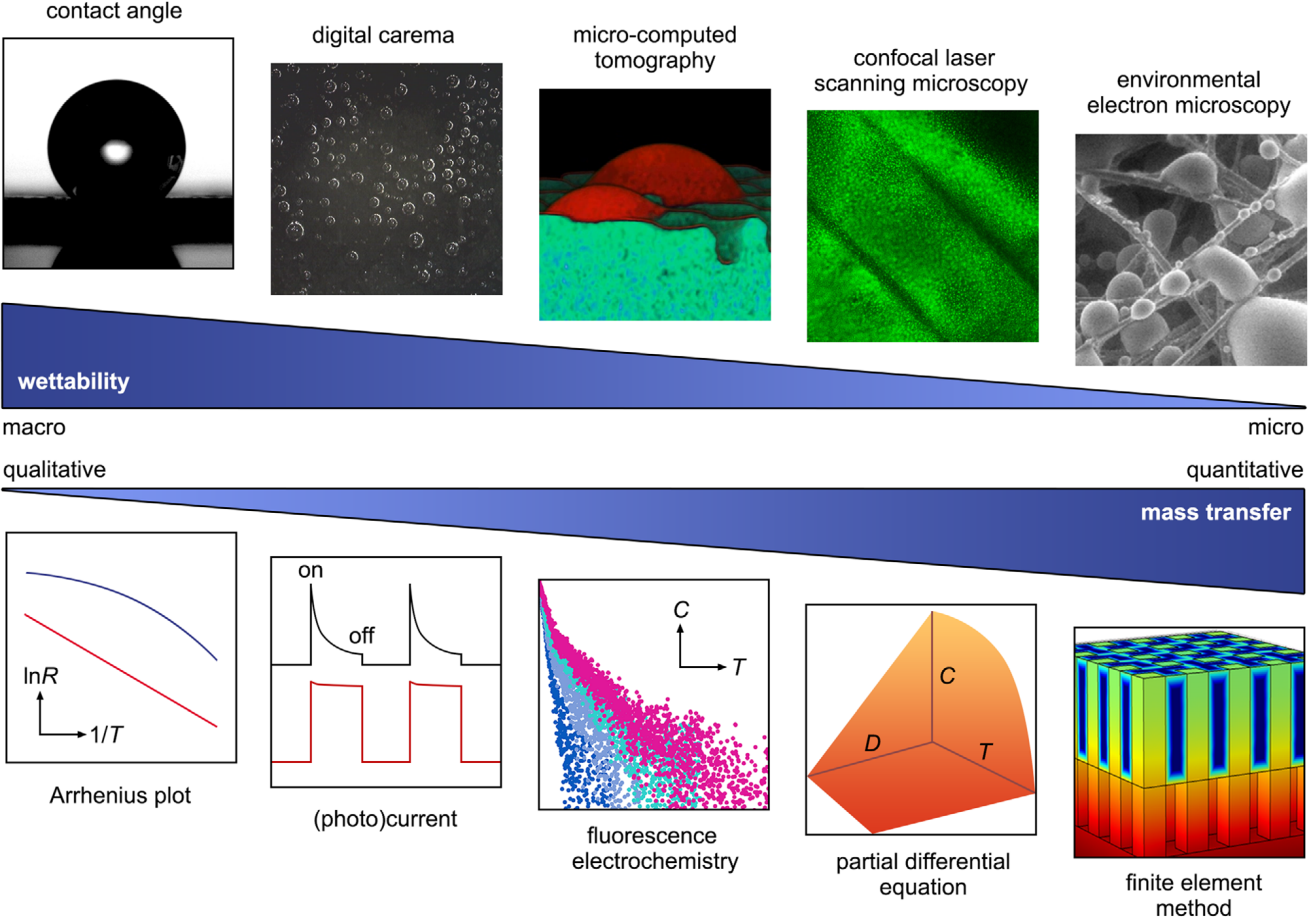
Experimental and computational strategies applied for triple‐phase wettability and mass transfer characterization in the order of spatial resolution and analysis accuracy. Micro‐computed tomography image is reproduced with permission.^[^ ^80^ ^]^ Copyright 2016, Wiley‐VCH. Environmental electron microscopy image is reproduced with permission.^[^ ^59^ ^]^ Copyright 2015, American Chemical Society

In this review, we summarize ten types of experimental and computational methods that have been or are expected to be applied in characterizing the interfacial wettability and the mass transfer process in triple‐phase catalysis (Figure 1). The discussion mainly contains widely investigated photo‐ and electrocatalytic reactions involving gas and water molecules as reactants and/or products. We prospect the advantages and limitations of each characterization method, with particular attention to their expansibility in the future development of triple‐phase catalysis.

## 2 CHARACTERIZATIONS OF THE INTERFACIAL WETTABILITY

The overall reaction process on the surface of heterogeneous catalysts typically includes (1) reactants transport; (2) reactants adsorption; (3) intermediates conversion; (4) products desorption; and (5) products transport. The interfacial structure plays a key role in determining the efficiency of the catalytic reaction. For triple‐phase catalysis, the interfacial structure refers to structures of both the catalyst and the gas–liquid–solid interface.^[^ ^23^ ^]^ The former can be well‐controlled during material preparation procedures and characterized by conventional material characterization technologies, such as X‐ray spectrum and electron microscopy. While the latter is highly dependent on the wettability and wetting state of the catalyst and is very sensitive to the reaction environment, thus providing new opportunities and challenges for the characterization.^[^ ^27^, ^28^ ^]^ Beginning with contact angle (CA) measurements, the characterizations of triple‐phase interfaces are discussed in the order of spatial resolution from macro to micro in the following sections.

### 2.1 CA measurements

**FIGURE 2:**
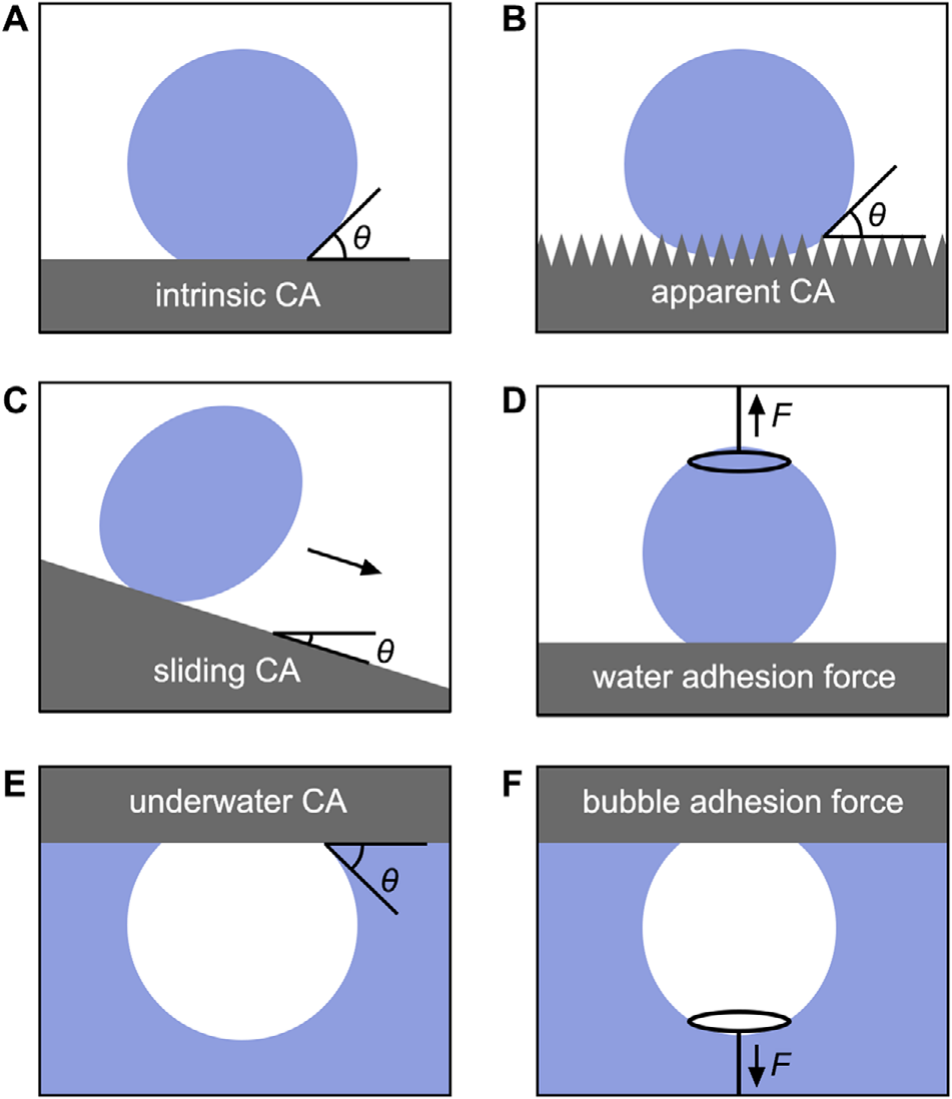
Schematic illustration of different types of contact angle (CA) measurements for triple‐phase wettability characterization. Gas‐consuming reactions often use (A) intrinsic CA, (B) apparent CA, (C) sliding CA, and (D) water adhesion force. Gas‐evolving reactions often use (E) underwater CA, and (F) bubble adhesion force

CA is a fundamental parameter to analyze the wettability of a triple‐phase interface.^[^ ^29^ ^]^ It is a universal method to observe the wettability of nanostructured catalysts due to their simplicity and compatibility. The result can reflect the intrinsic or apparent wettability for smooth and rough materials, respectively.^[^ ^30^ ^]^ CA measurements can be sorted as air phase water droplet and underwater gas bubble measurements (Figure 2). Both have been frequently used in recent years to study the wettability of gas‐consuming and gas‐evolving reaction systems.

#### 2.1.1 Water CAs for gas‐consuming reactions

Water droplet CAs are often measured in gas‐consuming electrocatalysis since most electrochemical energy conversion and storage processes involve interface contact problems between electrodes, electrolytes, and gas reactants, such as O_2_/CO_2_/CO reduction, fuel cells, and metal‐air batteries.^[^ ^31^, ^32^, ^33^, ^34^, ^35^ ^]^ For example, Cui and co‐workers tested CAs to show the wettability of surface‐modified polyethylene membrane electrodes, which were used for the artificial lung pouch‐like triple‐phase CO_2_ and O_2_ reduction.^[^ ^36^, ^37^ ^]^ Air plasma treatment can be used for the hydrophilic modification of carbon‐based electrodes by introducing surface oxygen‐containing groups.^[^ ^38^ ^]^ A gradually increased hydrophilicity with CA decreased from 134° to 21° was achieved for gold/carbon nanoparticle electrocatalysts treated with air plasma for 2.5 min. On the contrary, surface ligands modification could deliver hydrophobic properties with different degrees according to the ligand's composition and coverage.^[^ ^39^, ^40^ ^]^ For copper dendrite nanoarray electrode, 1‐octadecanethiol modification dramatically increased its CA from 17° to 153°.^[^ ^41^ ^]^ The better hydrophobicity contributed to the formation of abundant triple‐phase interfaces and reduced CO_2_ reduction overpotential.^[^ ^42^ ^]^ CA measurement also provides valuable information about the stability of the wettability after electrocatalysis, which is an essential parameter to evaluate the anti‐flooding property of gas diffusion electrodes.^[^ ^43^ ^]^ CA of pristine copper catalyst on gas diffusion electrode significantly decreased from 144.2° to 55.4° after 2 h CO_2_ electrolysis, indicating its poor interfacial stability. After mixing the copper catalyst with hydrophobic Teflon particles, the electrode maintained a stable CA at above 140° after the same electrolysis.

For photocatalysis, most semiconductor nanomaterials such as titanium oxide and carbon nitride are hydrophilic and show a water CA close to zero.^[^ ^44^ ^]^ It means that the pristine nanoparticles of these materials cannot form stable triple‐phase interfaces in aqueous media. Substrate immobilization and surface modification strategies have been applied to build robust gas–liquid–solid interfaces for photocatalytic reactions. Feng and co‐workers immobilized titanium oxide nanoparticles on a porous substrate layer and placed the substrate to the air–water interface with titanium oxide side contact to the water phase.^[^ ^45^ ^]^ The superhydrophobic property of the porous substrate (CA = 155 ± 2°) prevented the infiltration of water, thus allowing the formation of abundant triple‐phase interfaces over titanium oxide nanoparticles. A couple of the following works proved the advantages of the triple‐phase photocatalytic system in achieving higher reactivity, apparent quantum yields, and photothermal effects than the double‐phase system without a gas‐phase connection.^[^ ^46^, ^47^, ^48^ ^]^ Besides, the CA of carbon nitride nanosheets can be modified from near zero to 120° by grafting with 1*H*,1*H*,2*H*,2*H*‐perfluorodecanethiol.^[^ ^49^ ^]^ The hydrophobic surface could adsorb CO_2_ bubbles when the carbon nitride nanosheets were immersed in a CO_2_‐saturated aqueous phase, thus leading to the formation of triple‐phase interfaces and improved photocatalytic CO_2_ reduction activity.

Recently, interfacial wettability has also attracted attention in low‐temperature thermocatalytic processes, including methane oxidation, CO hydrogenation, and water–gas shift reactions.^[^ ^50^, ^51^, ^52^ ^]^ One common characteristic of these reactions is the involvement of H_2_O/H_2_O_2_ as reactants, intermediates, or products. For instance, coating molecular sieves with an organosilanes hydrophobic layer could confine the in situ generated hydrogen peroxide, contributing to enhanced methanol productivity.^[^ ^50^ ^]^ Similarly, a significantly increased water CA from ∼40° to ∼138° was obtained for surface‐modified iron catalysts.^[^ ^51^ ^]^ The improved hydrophobicity protected the catalyst surface from water generated during CO hydrogenation, thus achieving an olefin yield of up to 36.6% with suppressed side reactions related to water.

#### 2.1.2 Underwater CAs for gas‐evolving reactions

For gas‐evolving reactions, such as oxygen evolution reaction and hydrogen evolution reaction, gas bubbles are generated and detached from the surface of catalysts immersed in aqueous media. This stimulates the observation of underwater triple‐phases, where small gas bubbles rather than continuous gas phases are exposed to the catalyst. The underwater gas bubble CA measurement for hydrogen evolution reaction was firstly reported in 2014 to reveal the superaerophobic behavior of a nanostructured electrode.^[^ ^53^ ^]^ The vertically oriented MoS_2_ nanosheet array on the electrode provided more electrochemical active surface area and, in particular, showed a substantially larger gas bubble CA (153.6°) than the flat electrode (135.2°). The superaerophobic behavior allows the immediate departure of small hydrogen bubbles from the surface of the electrode, therefore contributing to efficiently exposed active sites for the following cathodic reaction.^[^ ^54^ ^]^ This property is significant for improving the energy efficiency and electrode stability of a water splitting system operated at industrial level current densities.

Beyond gas‐evolving reactions represented by water splitting, the CA measurement of underwater gas bubbles is also useful to evaluate the interfacial wettability of gas‐consuming reactions, especially for those underwater aerophilic triple‐phase systems.^[^ ^55^ ^]^ The underwater triple‐phase system typically consists of hydrophobic catalysts with porous or array nanostructures. Teflon‐modified gas diffusion electrode typically shows a gas bubble bursting effect during underwater CA measurement (CA ≈ 0°), indicating that gas pockets would fill in the pores or intervals of the nanoarrays.^[^ ^55^ ^]^ In this case, gaseous reactants can directly diffuse to the surface of catalysts until all reactants in gas pockets are consumed up. Nevertheless, constructing an underwater triple‐phase system is a trade‐off process. More gas pockets surrounding the catalyst indicate less connection to the water phase, which was evidenced from carbon and copper nanoarray electrodes for oxygen and CO_2_ reduction reactions, respectively.^[^ ^31^, ^56^ ^]^ Besides, an aerophilic surface modification toward catalysts is often at the cost of covered active sites and increased internal resistance. Theoretically, a catalyst with moderate wettability is desirable to optimize the contact of both gas and liquid phases and provide abundant triple‐phase interfaces. CA measurement alone, however, can hardly give detailed structural information about the abundance and the shape of interfaces, stimulating the extension and collaboration with other characterizations.

#### 2.1.3 Extension

Adhesive force is a widely used extension of CA measurements to analyze the relative bonding strength of catalysts to water and gas phases. The adhesive force of a water droplet or a gas bubble to a solid surface is ranged from near zero to hundreds of micro‐newton.^[^ ^54^, ^57^ ^]^ For example, a moderate adhesive force to a gas bubble indicates the formation of a stable triple‐phase interface, which is suitable for gas‐consuming reactions in the liquid phase. On the contrary, a small bubble adhesive force is required for gas‐evolving reactions to keep active sites exposed to the liquid reactants. According to reported literature in catalysis, the results of adhesive force are always in good agreement with the CA measurements, where a big CA toward droplet/bubble refers to a weak adhesive force and vice versa. A more intriguing reverse combination, which is often observed in nature (fish: hydrophilic with weak adhesion; wall gecko: hydrophobic with strong adhesion), has yet to be discovered in triple‐phase catalysis.^[^ ^30^, ^58^ ^]^ It should be noted that the adhesive force measurement has a limitation in the repeatability due to the inconsistent experimental details, such as the structure of the pallet, and the initial extrusion strength of the droplet/bubble, therefore cannot be directly used for quantitative study and comparison between different works.

For a superwetting system, where regular CA and adhesive force measurements can only give limited information, dynamic CA hysteresis measurements, including advancing, receding, and sliding CAs, are often used as alternatives to characterize the interfacial wettability.^[^ ^59^ ^]^ In addition, owing to the simplicity and compatibility, CA measurements can be used as an operando technique to investigate the wettability during catalytic reactions. Although few examples can be found from literature up to date, one can easily find the feasibility of adding a series of variables to the measurement, such as light intensity, wavelength, bias potential, time, temperature, pressure, and gas/liquid composition, thus greatly extending the innovation of this method in triple‐phase catalysis.

### 2.2 Optical microscopy

Optical microscopy is an optical instrument that uses the optical principle to magnify and image material's microstructures that cannot be distinguished by human eyes. For triple‐phase catalysis, it is typically used to observe microscale gas bubbles and local liquid phase connected to catalysts and can be divided into ultrafast digital cameras and confocal laser scanning microscopy (CLSM). The resolution of optical microscopy ranges from micrometers to nanometers according to the light source selection and optical path setting.^[^ ^60^ ^]^ Although optical microscopy shows relatively lower resolution than electron microscopy, it offers faster response and better environmental compatibility that can be used to image triple‐phase interfaces under catalytic conditions.

#### 2.2.1 Digital camera

**FIGURE 3:**
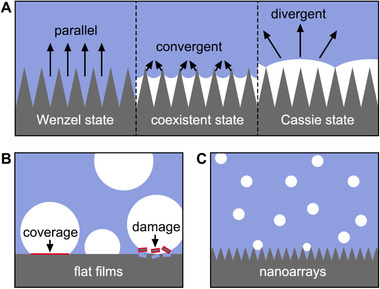
(A) Scheme of the wetting state characterization by optical imaging. A parallel reflection indicates a Wenzel state without a triple‐phase interface. A convergent reflection (bright region) implies a Cassie‐Wenzel coexistent state with the formation of abundant triple‐phase interfaces. A divergent reflection (dark region) indicates a Cassie state that gas pockets fully cover the surface of catalysts. (B) Gas bubble detachment over flat film electrode. (C) Gas bubble detachment over nanoarray electrode

Digital camera imaging has been applied to reveal the wetting state of nanostructured electrodes (Figure 3A).^[^ ^57^ ^]^ The wetting state of a rough surface can be classified as three models: (1) Cassie model: non‐wetted contact between the liquid and the rough surface; (2) Wenzel model: fully wetted contact between the liquid and the rough surface; and (3) Cassie‐Wenzel coexistence model: an intermediate state between Cassie and Wenzel models. For electrocatalytic oxygen reduction, Liu and co‐workers revealed the different wetting states of the surface‐modified Pt/Si array cathode by comparing the different reflection contrast (parallel, convergent, and divergent) from top‐view optical images.^[^ ^57^ ^]^ The observation demonstrates the structure of the triple‐phase catalytic interface and correlates well with the CA and adhesive force measurements.

Unlike the above static optical imaging for underwater triple‐phase systems, in which exotic gas bubbles are used for the measurement, dynamic imaging is useful to detect the in situ generation and release of gas bubbles on the surface of catalysts.^[^ ^61^ ^]^ It gives intuitive evidence about the interaction between as‐generated bubbles and electrodes in gas‐evolving reactions. Generally, the average diameter of the released bubbles increases with hydrophilicity reduction. The nanostructure of an electrode can also largely affect the bubble departure diameter. For noble metal electrodes, such as gold and platinum, the bubble departure diameter could reduce from about 500 μm to less than 50 μm by adjusting the morphology of the electrode from flat films to nanoarrays.^[^ ^62^, ^63^ ^]^ The unreleased bubbles on the surface of the electrode will result in coverage of active sites and reduced apparent electrochemical activities. Besides, the strong detachment force of big bubbles often leads to the damage of the electrode (Figure 3B,C).

#### 2.2.2 CLSM

CLSM is one important structured illumination microscopy developed to achieve optical superresolution.^[^ ^64^ ^]^ CLSM typically shows higher axial and lateral resolution than conventional wide‐field optical microscopy mainly due to shielding the noise interference from out of the focal plane. It has been widely used in bio‐related investigations, such as cell imaging, drug delivery, and cancer therapy.^[^ ^65^, ^66^, ^67^, ^68^ ^]^

**FIGURE 4:**
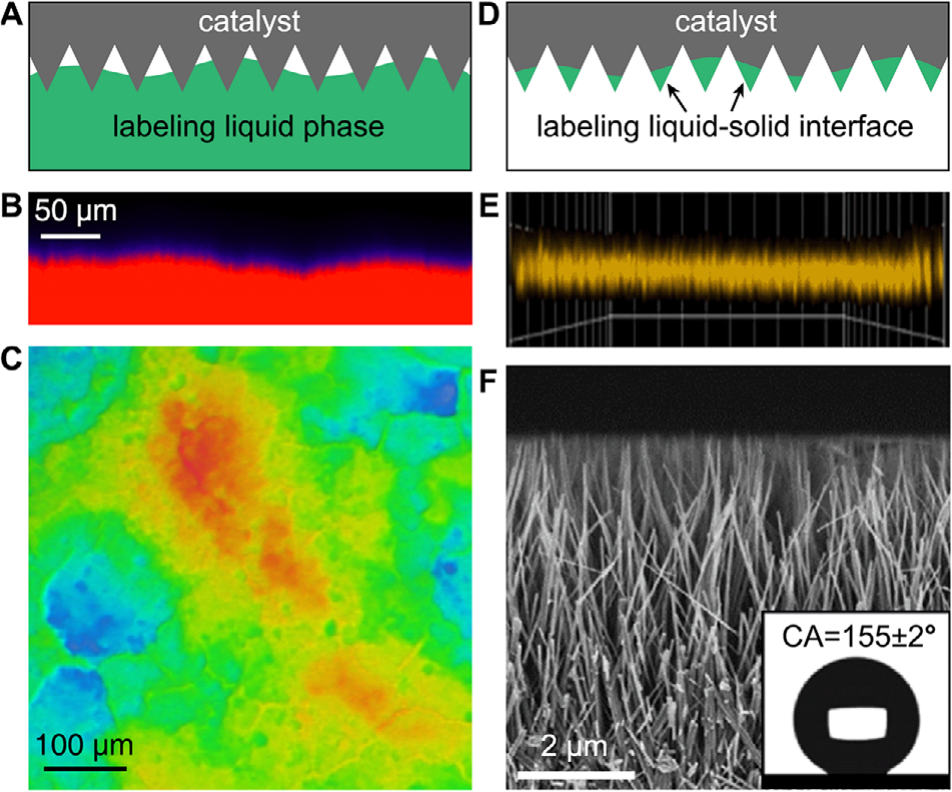
CLSM for imaging triple‐phase catalytic interfaces. (A) Schematic illustration of labeling liquid phase with fluorescent dyes for CLSM. This method shows advantages in online testing the interfacial wettability during catalytic reactions. (B) 2D cross‐sectional image of an air–water–TiO_2_ interface. Bright red region refers to Rhodamine‐6G labeled water phase. (C) Corresponding 3D reconstruction image of the triple‐phase interface. Reproduced with permission.^[^ ^125^ ^]^ Copyright 2020, Elsevier. (D) Schematic illustration of labeling liquid–solid interface with fluorescent dyes for CLSM. This method shows advantages in measuring the penetration depth of water into catalyst layers. (E) Side image of Rhodamine B labeled TiO_2_ nanowire arrays after drying the water phase. (F) Cross‐sectional SEM image of the TiO_2_ nanowire arrays. Reproduced with permission.^[^ ^47^ ^]^ Copyright 2020, American Chemical Society

The features of high resolution, quick response, excellent environmental compatibility (tolerance to liquids and gases), and selective imaging of fluorescent molecules labeled areas allow the possible application of CLSM in other fields, including the imaging of patterned colloidal nanocrystals.^[^ ^69^ ^]^ Very recently, CLSM showed advantages in revealing the triple‐phase catalytic interfaces.^[^ ^38^ ^]^ Different from the above applications, most catalysts show poor photoluminescence and light transparency, therefore cannot be directly observed from both the dark‐field fluorescent imaging and bright‐field optical imaging. As an alternative, the liquid phase can be labeled with various fluorescent molecules to adapt to different liquid environments (Figure 4A). The imaging of patterned liquid phase can reflect the shape of gas–liquid and liquid–solid boundaries, thus providing valuable information to predict the wetting state of nanostructured triple‐phase interfaces.

Alkali‐tolerant fluorescein is suitable for labeling alkali electrolytes frequently used in electrocatalysis.^[^ ^70^ ^]^ Through 2D cross‐sectional and 3D reconstruction imaging, we can illustrate the electrolyte's penetration depth into the pores of the catalyst layer. Therefore, a wetting state characterization of triple‐phase catalytic interfaces can be achieved, which shows higher spatial resolution than the aforementioned optical imaging by digital cameras. CLSM has also been used for photocatalysis to image the wetting state of semiconductors immobilized on gas‐diffusion layers (Figure 4B,C). It helps to strengthen the reliability of wettability analysis, which is often questionable only based on CA measurements. A moderate wettability with a Cassie‐Wenzel coexistence model has been demonstrated to be essential for carbon dioxide and oxygen reduction reactions, mainly owing to the maximized triple‐phase contact points.^[^ ^38^, ^57^ ^]^ The latest understanding revealed that for a gas‐consuming reaction, gas reactants reach the catalyst's surface by dissolution and diffusion through the local water phase rather than being directly converted at the triple‐phase contact points.^[^ ^25^ ^]^ It indicates that a proper wetting state can also promote the interfacial mass transfer of gas and liquid reactants (more discussions can be found in Section 3).

CLSM is currently the state‐of‐the‐art optical imaging technology for investigating triple‐phase interfaces, and there is still much room for improvement. The coupling of optical microscopy with other microscopic technologies is a good direction and will be briefly introduced in Section 2.4. Beyond off‐line characterization, developing operando CLSM imaging is critical to observe the change of a triple‐phase interface during catalytic reactions. In addition, similar to the colorful imaging of cell structures, labeling the three phases with different fluorescent dyes is believed to be helpful to further enhance the capability of CLSM for triple‐phase interface imaging. However, it is challenging because of the difficulty in labeling gas and catalyst phases.

### 2.3 X‐ray micro‐computed tomography 3D imaging

Micro‐computed tomography (micro‐CT) 3D imaging was firstly developed in the early 1980s to study bone architecture.^[^ ^71^ ^]^ It shows a spatial resolution ranging from 1 to 50 μm, which is much higher than the original CT imaging technology and shows wide applications in diagnostic imaging, such as bone microarchitecture, phenotype evaluation, and vascular imaging.^[^ ^72^ ^]^ The 3D reconstructed images for micro‐CT could give the specimens’ spatial distribution maps, including their internal structures, which is difficult for optical and electron microscopies. Many types of materials (inorganics, soft tissues, and liquids) can be directly observed and distinguished with high signal contrast from micro‐CT images.^[^ ^73^ ^]^ These advantages allow micro‐CT to investigate the 2D/3D structures of both catalyst/substrate interfaces and the triple‐phase interfaces.

**FIGURE 5:**
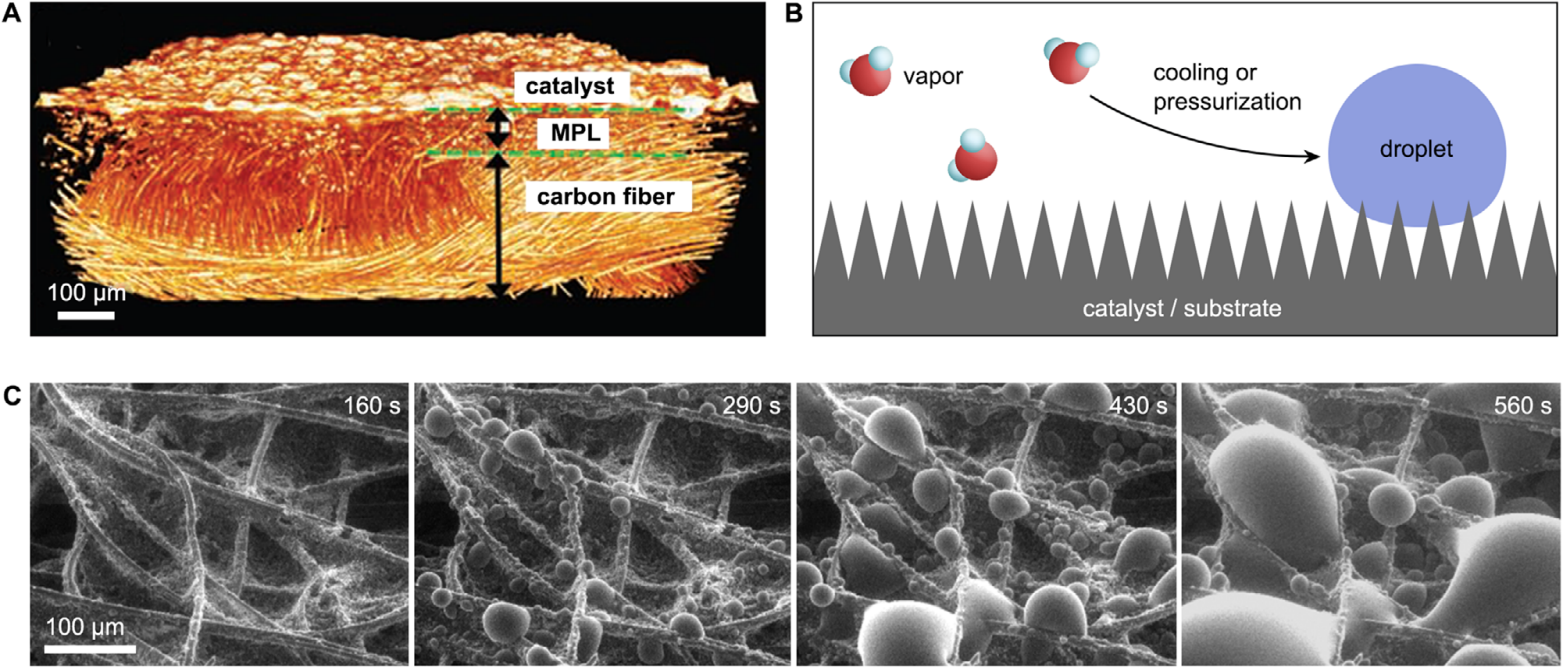
(A) Micro‐CT 3D tomographic virtual model of air‐brushed Pt/C nanoparticles on gas diffusion cathode. MPL, microporous layers. Adapted with permission.^[^ ^78^ ^]^ Copyright 2013, WILEY‐VCH. (B) Schematic illustration of microscale wettability characterization by ESEM. A programmed cooling (or pressurization) process in the specimen chamber allows the condensation of low‐pressure water vapor into water microdroplets on catalysts or substrates. (C) ESEM images of water condensation on hydrophobic gas diffusion electrode by increasing the vapor pressure for 160, 290, 430, and 560 s. Reproduced with permission.^[^ ^59^ ^]^ Copyright 2015, ACS Publication

The interface between catalytic active sites and supporting substrates, such as metal/oxide interface in thermal catalysis, catalyst/electrode in electrocatalysis, and co‐catalyst/semiconductor in photocatalysis, have demonstrated to be a key factor in determining the overall catalytic performance.^[^ ^74^, ^75^, ^76^ ^]^ For example, the contact between the electrocatalyst and electrode substrate could significantly affect the conductivity, electrochemical active surface area, and interfacial mass transfer of the system.^[^ ^77^ ^]^ Cross‐sectional electron microscopy is often used to characterize the structure of catalyst/electrode interfaces. However, this method requires complex sample preparation processes and inevitability changes the interface structure during preparation. Using micro‐CT, we can collect the nanostructure of the catalyst layer, electrode, and their interface through 2D cross‐sectional and 3D reconstructed images without damaging the material (Figure 5A).^[^ ^78^, ^79^ ^]^ The structure of Pt/C and Ag catalyst layers on gas diffusion electrodes has been revealed by Kenis and co‐workers. They proved that a catalyst layer with a uniform catalyst distribution (closely combined with the substrate without particle agglomeration) possessed the optimized performance for fuel cells and CO_2_ electroreduction.

Since the attenuation coefficients of X‐ray signal is highly dependent on the composition of detected materials, micro‐CT could reveal the relative position and interfacial contact between each phase (liquid–solid interface, for example).^[^ ^72^ ^]^ The liquid–solid boundary can be clearly distinguished due to the contrast between liquid and solid phases, therefore helping to observe the microscale wetting state of the interface. In 2016, Jiang and co‐workers reported an anisotropic wetting state of ethylene glycol droplets at a biomimetic triple‐phase interface based on micro‐CT 3D reconstruction images.^[^ ^80^ ^]^ They observed the unidirectional transportation of droplets on an artificial peristome surface of Nepenthes alata. This suggests the possibility of using micro‐CT to analyze a gas–liquid–solid interface for a catalytic system. However, only liquids with high boiling points (e.g., ethylene glycol) were used for this technique. The detection of aqueous solution as the most investigated liquid phase in catalysis has yet to be reported.

In brief, micro‐CT is a powerful imaging technology with a comparable spatial resolution to confocal optical microscopy. Currently, its application is mostly focused on solid substances, and its potential in revealing the wettability of triple‐phase interfaces is underestimated. Its imaging principle determines that both liquid and solid phases can be observed without labeling or other pre‐treatment, which provides a possibility to online capture the microscale wettability and wetting state of a catalytic interface.

### 2.4 Environmental scanning electron microscopy

Environmental scanning electron microscopy (ESEM) is a high‐resolution imaging tool to investigate the wettability of nanostructured materials.^[^ ^81^ ^]^ Through a programmed cooling (or pressurization) stage, the water vapor in the specimen chamber can be condensed to form small water droplets on substances (Figure 5B).^[^ ^82^ ^]^ However, only water vapor with pressure below 3 kPa is allowed, which severely limits the application of this imaging technology in the analysis of triple‐phase interfaces.^[^ ^83^ ^]^ Most ESEM used in catalysis investigations reported so far is to observe the condensed water microdroplet on carbon fiber, which is the building block of gas diffusion layer widely used in CO_2_ electrolysis, fuel cells, and metal‐air batteries.^[^ ^84^ ^]^ For example, Moon and co‐workers demonstrated by ESEM that coating commercialized gas diffusion layers with a hydrophobic nanolayer can significantly reduce the water condensation rate when exposed to a humid environment (Figure 5C).^[^ ^59^ ^]^ Besides, ESEM shows special advantages to investigating the flooding effect of porous gas diffusion substrate, which is deemed as a major issue in reducing the mass transfer efficiency and operational stability of triple‐phase catalysis.^[^ ^85^ ^]^ Nonetheless, using ESEM to determine the microscale water CA and wetting state of catalytic nanostructures has received little attention.

Ionic liquids, a significant kind of non‐molecular compounds in the liquid state that has been investigated for high‐performance electrocatalysis, typically have a much higher boiling point than water.^[^ ^86^, ^87^ ^]^ This feature makes ionic liquids more feasible than water for ESEM imaging. Ionic liquids can be divided into hydrophilic and hydrophobic according to their compositions, making the interfacial structure between them and catalysts an exciting but challenging research topic.^[^ ^88^ ^]^ A Cassie wetting state of 1‐hydroxyethyl‐3‐methylimidazolium tetrafluoroborate ionic liquids on –CF_3_ terminated nanostructured surfaces have been successfully observed by ESEM, which sheds light on the characterization of the gas‐ionic liquids‐electrode interface in non‐aqueous electrocatalysis.^[^ ^89^ ^]^

A combination of SEM and optical microscopy can make full use of the advantages of the two techniques (high resolution and excellent environmental compatibility) and has been applied for the spectrum analysis of individual nanocrystals. Such combination requires the alignment of the sample, which is typically achieved by orientation correction to a marked substrate.^[^ ^90^ ^]^ By coupling SEM with optical microscopy, it is possible to get high‐resolution images of nanocatalysts and the corresponding triple‐phase interfaces. For example, an oil–silica–water triple‐phase nanostructure can be well‐confirmed by combining SEM (imaging hollow silica nanospheres) with dark‐field optical microscopy (imaging oil nanodroplets).^[^ ^91^ ^]^ The nanostructure acts as an oxygen nanocarrier and shows enhanced interfacial oxygen diffusion for biocatalytic oxidation of glucose, choline, lactate, and sucrose. Coupling with CLSM has also been demonstrated to be useful in determining the penetration depth of the water phase into the photocatalytic nanoarrays (Figure 4D–F).^[^ ^47^ ^]^ By evaporating the fluorescent‐labeled liquid droplet on the catalyst, researchers can clearly illustrate the thickness of the fluorescent layer (i.e., the penetration depth of the water phase) and its relative position to the photocatalytic nanoarrays through the comparison between SEM and CLSM images.

## 3 CHARACTERIZATION OF THE INTERFACIAL MASS TRANSFER

For a specific triple‐phase catalytic system, its interfacial wettability and molecular mass transfer are the two sides of a coin. Mass transfer characterization helps build the relationship between interfacial structures and catalytic activities, thus guiding the design of advanced catalytic systems. Both experimental and computational methods have been developed to investigate the interfacial mass transfer process. The following sections introduce three kinds of experimental measurements and two kinds of computational simulations and discuss their capabilities in detecting the mass transfer process at triple‐phase interfaces.

### 3.1 Experimental measurements

#### 3.1.1 Arrhenius plot

The Arrhenius plot shows the temperature‐dependent reaction rate of chemical processes, which is typically used to evaluate apparent activation energies (*E*_a_).^[^ ^92^ ^]^ For most chemical reactions, the logarithm of the reaction rate is linearly correlated with the reciprocal of temperature, indicating a kinetic control regime. The *E*_a_ can be measured from the slope of the fitted curve −*E*_a_/*R*, where *R* is the gas constant. However, a non‐linear relationship also exists in some conditions. For example, a convex curve was detected for dimethyl oxalate hydrogenation in a porous nanocage reactor.^[^ ^93^ ^]^ The curve's curvature gradually reduced with the decrease of the size of the nanocage, accompanied by a reduced dimethyl oxalate diffusion flux. Therefore, the downward deviation from the Arrhenius linear relationship can be understood from a diffusion control regime that the accelerated activity at higher temperatures would induce a more severe diffusion limitation and cause the curve to bend.

This phenomenon is rarely seen in gas–solid catalysis at low temperatures due to the fast mass transfer of gas reactants in the gas phase.^[^ ^94^ ^]^ But for photo‐ and electrocatalytic reactions involving slow mass transfer processes of gas molecules in liquid phases, the Arrhenius plot is a useful tool to determine the rate‐determining step of the reaction. Electrochemical acetylene reduction to ethylene has recently become a hot research topic owing to its advantages in removing trace amounts of acetylene from ethylene gas feed compared to the conventional hydrogenation route.^[^ ^95^, ^96^, ^97^ ^]^ For a triple‐phase electrochemical flow‐cell, an Arrhenius linear relationship was observed between the logarithm of the reaction rate and the reciprocal of temperature. However, a double‐phase control system immersing the electrode into an acetylene‐saturated electrolyte showed a non‐linear relationship. Interestingly, a concave curve was formed that reaction rate was primarily unchanged at temperatures between 5 and 15℃. This phenomenon was deemed as an abnormal diffusion‐controlled regime because of the higher acetylene solubility in the electrolyte at lower temperatures.

Therefore, the Arrhenius plot or the relationship between reaction rates and temperatures is one of the few experimental approaches for qualitatively investigating the interfacial mass transfer process. It can be used to make a preliminary judgment on the mass transfer process as the potential rate‐determining step of the reaction.

#### 3.1.2 General electrochemistry

The mass transfer process of reactants and products on the surface of electrode has been investigated for decades based on electrochemical measurements.^[^ ^98^, ^99^, ^100^ ^]^ For example, electrochemical impedance spectroscopy (EIS) can give semi‐quantitative interfacial mass transfer information of an electrochemical system.^[^ ^101^ ^]^ Nyquist plots detected from EIS typically involve high‐frequency and low‐frequency regions. The high‐frequency region reflects the electrode's internal resistance and charges transfer resistance, while the low‐frequency is linked to the diffusion behavior of electrolyte ions at catalytic interfaces. For example, a porous electrode with higher hydrophilicity (or thicker catalyst layers exposed to electrolyte) typically shows a higher low‐frequency resistance due to the less diffusivity of ions into the interlayer of the electrode. The relaxation time constant obtained from the Nyquist plots can reflect how fast ions can transport inside the porous electrode.^[^ ^102^, ^103^ ^]^ Hydrophobic porous electrode processes shorter relaxation time constant than the hydrophilic one because of the smaller electrolyte penetration and apparent viscosity at electrolyte/electrode interfaces.^[^ ^104^ ^]^ But it is still challenging to establish an appropriate model to fix the EIS results and give accurate meaning to the Nyquist plot in triple‐phase electrocatalysis. Besides, the EIS measurement is mainly used for charge carrier transportation in electrochemical energy storage. It should be noted that the dissolution and consumption of gas reactants at triple‐phase interfaces can also affect the interfacial transport of electrolyte ions. Therefore, researchers should deserve more attention to the EIS measurement in evaluating the interfacial mass transfer property in triple‐phase electrocatalysis.

**FIGURE 6:**
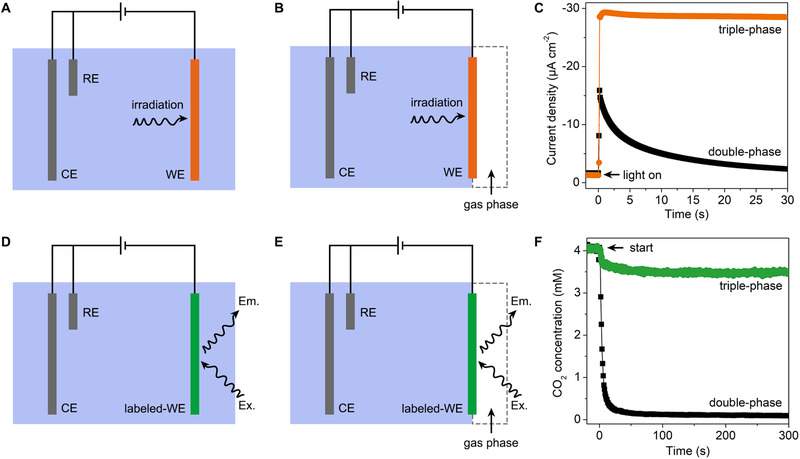
Scheme of photocurrent measurement for (A) double‐phase and (B) triple‐phase systems. (C) Oxygen reduction photocurrent tests over Au‐TiO_2_ photocathode. The triple‐phase system showed a higher and more stable photocurrent density than the corresponding double‐phase system. Reproduced with permission.^[^ ^107^ ^]^ Copyright 2021, Elsevier. Scheme of fluorescence electrochemical spectroscopy measurement for (D) double‐phase and (E) triple‐phase systems. (F) Electrochemical CO_2_ reduction over gold/carbon cathode labeled with CO_2_‐sensitive fluorescent molecules. The triple‐phase system showed a more stable interfacial CO_2_ concentration than the corresponding double‐phase system during electrolysis. Reproduced under the terms of the CC BY‐ND 2.0 license.^[^ ^38^ ^]^ Copyright 2020, Run Shi et al. WE, working electrode. CE, counter electrode. RE, reference electrode. Ex., excitation light. Em., emission light

Photocurrent test is used for evaluating the charge separation of a photocatalyst from either the steady‐state photocurrent density or the transient photocurrent rise/decay curves.^[^ ^105^ ^]^ Typically, photocatalysts were grown or immobilized on conductive glass substrates for the measurement (Figure 6A).^[^ ^106^ ^]^ However, the working electrode immersed in the electrolyte can only provide a liquid–solid microenvironment and gives limited information for the interfacial mass transfer process of gas‐consuming reactions. This obstacle can be overcome using a hydrophobic porous substrate instead of a glass plate (Figure 6B). Through the coating of Au‐TiO_2_ nanoparticles on the surface of a gas diffusion electrode, Zhang et al. evaluated the interfacial oxygen mass transfer in triple‐phase photocatalytic aerobic oxidation reactions.^[^ ^107^ ^]^ The system exhibited a stable photocurrent density during each chopped ultraviolet irradiation, which can be ascribed to the significantly promoted interfacial oxygen mass transfer process (Figure 6C). On the contrary, a significant decay of photocurrent density within every irradiation period was observed for a double‐phase control system, indicating oxygen exhaustion‐induced charge recombination. One should note that oxygen evolution reaction is preferred on n‐type semiconductors, which means that a proper bias potential should be applied to most metal oxide photocatalysts for investigating oxygen reduction reactions (or other gas‐consuming reactions).^[^ ^108^, ^109^ ^]^ It is believed that the method is also applicable to investigate the mass transfer process in electrocatalysis without illumination by setting an appropriate intermittent bias to the working electrode.

EIS and photocurrent are useful tools to comprehensively investigate the charge separation and interfacial mass transfer processes of a triple‐phase photocatalytic system. But it can only give rough qualitative results about whether the reaction is a mass transfer controlled process. The quantification of the interfacial mass transfer process relies on the correlation between the current density and the interfacial concentration of reactants, which will be discussed in the following section.

#### 3.1.3 Fluorescence electrochemical spectroscopy

From the interfacial mass transfer equation (Equation (1)), one can find that the mass transfer flux (*N*) is a function of mass transfer coefficient (*k*) and the concentration difference between the bulk phase (*C*_0_) and the interface (*C*_i_).^[^ ^110^ ^]^

(1) $$N=k\left(C_{0}-C_{i}\right)$$

The reactant concentration in the bulk phase is a known constant. Therefore, mass transfer flux and interfacial concentration should be measured to quantitatively evaluate the interfacial mass transfer coefficient of a catalytic system. For heterogeneous catalysis, it is reasonable to make an approximation that the reactant's interfacial mass transfer flux equals its consumption rate per unit area to reach a dynamic reaction equilibrium. Generally, the reactant consumption rate is proportional to the (photo)current and other detectable reaction signals. Therefore, quantifying the mass transfer coefficient as the intrinsic property of a catalytic system becomes possible by further measuring the interfacial concentration of the reactant. Recently, fluorescent labeling has been used for the in situ detection of interfacial CO_2_ concentration during CO_2_ electroreduction reaction (Figure 6D–F).^[^ ^38^, ^111^, ^112^ ^]^ A gas diffusion electrode labeled with CO_2_‐sensitive fluorescent molecules was equipped into fluorescence electrochemical spectroscopy (FES), of which the fluorescence intensity can be measured and converted to the interfacial CO_2_ concentration during the reaction. FES results demonstrated a stable interfacial CO_2_ concentration for the triple‐phase electrochemical CO_2_ reduction system at a current density of 100 mA cm^–2^. The CO_2_ mass transfer coefficients were determined to be 0.27 cm^–2^, which is over ten times higher than the double‐phase control system.

Theoretically, the FES measurement is suitable for other gas‐consuming reactions (oxygen reduction, nitrogen fixation, etc.).^[^ ^113^, ^114^, ^115^ ^]^ The only difference lies in selecting suitable fluorescent dyes for the specific reactant/product response. Experimentally detecting interfacial concentration and mass transfer coefficient is helpful to illustrate the interfacial molecular mass transfer property in triple‐phase catalysis and could give strong support to the widely applied theoretical simulations introduced in the next section.

### 3.2 Computational simulations

#### 3.2.1 Partial differential equations

CO_2_ as an important greenhouse gas and chemical feedstock has been intensively investigated for decades.^[^ ^27^, ^116^ ^]^ The chemical equilibrium between CO_2_, bicarbonate, and carbonate in the water phase often causes difficulties in precisely estimating the actual CO_2_ concentration for a series of photo‐ and electrocatalytic CO_2_ conversion reactions.^[^ ^117^ ^]^ More importantly, this issue cannot be ignored in triple‐phase CO_2_ electrolysis using an alkaline electrolyte, since the non‐faradaic consumption of CO_2_ will lead to severe CO_2_ loss and composition change of the electrolyte.^[^ ^118^ ^]^ Therefore, estimating the CO_2_ concentration at gas–electrolyte–cathode interfaces is important in understanding the overall process of CO_2_ mass transfer and chemical conversion.

**FIGURE 7:**
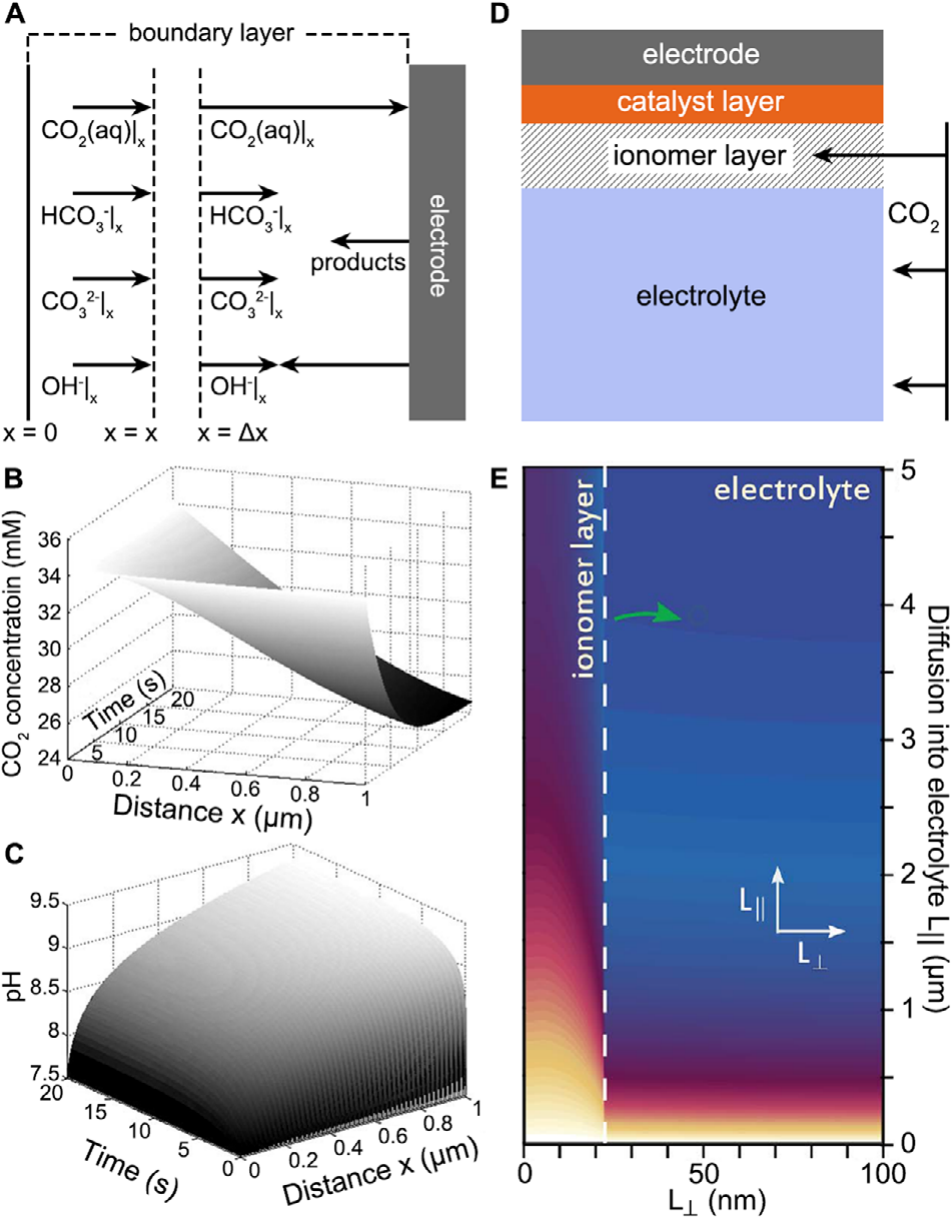
(A) A typical 1D boundary layer model used for solving partial differential equations in double‐phase electrocatalytic CO_2_ reduction. (B) Interfacial CO_2_ profile at a current density of 5 mA cm^–2^. (C) Interfacial pH profile at a current density of 5 mA cm^–2^. Adapted with permission.^[^ ^117^ ^]^ Copyright 2005, Springer Nature. (D) A scheme of 2D finite element model for simulating the interfacial CO_2_ diffusion property in the presence of an ionomer layer. (E) Modeled CO_2_ diffusion along with the catalyst layer in 5 M KOH electrolyte. The ionomer layer promotes the CO_2_ mass transfer across the catalyst's surface due to the improved effective diffusivity. L_||_, distance parallel to catalyst's surface; L_⊥_, distance perpendicular to catalyst's surface. Adapted with permission.^[^ ^121^ ^]^ Copyright 2020, AAAS

Partial differential equations (PDEs) take into account interactions (reaction and diffusion) between molecules and ions to model their interfacial mass transfer processes.^[^ ^119^ ^]^ Different from the above discussed experimental methods that the diffusion time and distance from the phase boundary cannot be precisely controlled, PDEs can provide time‐dependent concentration gradient as a function of diffusion distance from the catalytic interface. The concept of phase and interface is simplified as a one‐dimensional virtual space (the length is typically defined as the boundary layer thickness) with designated diffusion coefficients, reaction rate constants, and the initial concentration of solutes (Figure 7A). For CO_2_ electrolysis in KHCO_3_, time‐dependent concentration distribution from PDEs simulations revealed significantly different concentrations at electrolyte‐electrode interfaces than the bulk concentration (Figure 7B,C).^[^ ^117^ ^]^ As proved in many studies, the interfacial CO_2_ concentration is dramatically reduced during electrochemical reactions, mainly due to its low solubility and poor diffusion in the electrolyte. With the help of PDEs, Sinton et al. investigated the mass transfer influence of gas evolution and departure processes on electrode's surface to the effective diffusion layer thickness and the limiting CO_2_ reduction current densities.^[^ ^63^ ^]^ They found that the gas‐releasing effect (CO, H_2_, etc.) is a primary driving force for the interfacial mass transfer of dissolved CO_2_ molecules. The size of desorbed gas bubbles was highly dependent on the morphology of the electrode and can greatly affect the mass transfer efficiency and the CO_2_ reduction limiting current density.

When the electrolyte is switched to KOH, a well‐designed gas–electrolyte–cathode interface is required to facilitate CO_2_ supply and prevent non‐faradaic consumption by the OH^–^. PDEs played an irreplaceable role in estimating the interfacial pH and CO_2_ concentration during the reaction‐diffusion dynamic equilibrium process.^[^ ^120^ ^]^ The CO_2_ concentration in alkaline electrolytes decreases exponentially from the gas–liquid interface. Therefore, an ultrathin catalyst layer at the gas–electrolyte boundary was required to keep a relatively high local CO_2_ concentration in alkaline electrolyte and to achieve a high CO_2_ conversion selectivity with suppressed hydrogen evolution reaction at large current densities.^[^ ^120^ ^]^

The application of PDEs requires the determination of a series of initial conditions. However, the diffusion coefficient of CO_2_ in porous media (gas diffusion layer, polymer, etc.) cannot be directly obtained from the literature.^[^ ^107^, ^121^ ^]^ The collision between gas molecules and pore walls diminishes the effective diffusion coefficient of CO_2_ in nanoporous compared to CO_2_ in bulk. Bosanquet's effective diffusivity was employed to calculate the effective diffusion coefficient according to the Knudsen diffusion model approximation.

The exact solution of PDEs might not exist in complicated systems, such as the nanostructured 2D/3D triple‐phase catalytic interface, and we will discuss the alternative simulation method in the next section.

#### 3.2.2 Finite element method

Finite element method (FEM) is a modern computational method to find solutions to complex engineering problems. It is used to find approximate PDEs solutions of systems with complex geometry and boundary conditions by dividing the model into several subdivisions.^[^ ^98^, ^122^ ^]^ The complex system transforms into a series of simple solvable systems with relevant PDEs and dependent variables to simulate the mass transfer process at any point inside each element and over the whole system.

The diffusion coefficient of gas molecules in the gas phase is approximately 10^4^ and 10^9^ times higher than in liquid and solid phases.^[^ ^110^ ^]^ It means that constructing a gas phase highway directly to the surface of catalysts is the best way to solve the mass transfer issue for gas‐consuming reactions. However, the actual mass transfer process over triple‐phase interfaces is highly dependent on the wettability and nanostructure of the interface, often leading to a massive deviation from the ideal expectation.^[^ ^123^ ^]^ For example, the limiting CO_2_ reduction current density over the gas diffusion layer‐based triple‐phase system only increased about ten times compared to the double‐phase control sample.^[^ ^38^ ^]^ FEM provides a theoretical model to show the temporal and spatial distribution of a nanostructured boundary condition. Recently, Sargent et al. investigated the influence of an ionomer layer on the CO_2_ diffusion behavior across gas‐electrolyte‐catalyst triple‐phase interfaces (Figure 7D,E).^[^ ^121^ ^]^ FEM illustrated a significantly improved CO_2_ interfacial concentration during electrochemical reactions after coating catalyst with a hydrophobic ionomer layer. This improvement is mainly due to the ∼400 times higher effective diffusivity of CO_2_ in ionomer layers than CO_2_ in the electrolyte, and finally contributes to an increased limiting CO_2_ reduction current density from above 50 mA cm^–2^ to beyond 1 A cm^–2^. Gong et al. reported a nickel single‐atom catalyst with a 3D open‐pore nanostructure.^[^ ^124^ ^]^ FEM demonstrated the outstanding CO_2_ diffusion behavior over the open pore nanostructure compared to stacked graphene flakes and bulk carbon particles. The unobstructed CO_2_ mass transfer process in the liquid phase achieved a CO_2_ reduction current density outperforming most of the previously reported catalysts tested in H‐type cells.

Photocatalytic aerobic oxidation is another important reaction involving both oxygen and water as reactants. It shows a broad application in environmental catalysis, such as antibacterial, air purification, and organics degradation.^[^ ^125^, ^126^ ^]^ However, the oxygen mass transfer process during the reaction was ignored until the development of the first triple‐phase photocatalytic system in 2017.^[^ ^45^, ^127^ ^]^ The triple‐phase photocatalytic aerobic oxidation is supposed to have an efficient interfacial oxygen transfer and shows many advantages compared to nanoparticle‐dispersed liquid–solid systems: (1) suppressed charge recombination caused by the poor supply of oxygen; (2) broad oxygen concentration tolerance to keep a considerable reaction rate; (3) flow reaction feasibility. Recently, two FEM models have been built to simulate the local oxygen concentration over the photocatalyst layers immobilized at the air–water boundary and immersed in the water phase, respectively.^[^ ^107^ ^]^ The result showed that the local oxygen concentration dramatically decreased to near zero in the immersed double‐phase system, while the triple‐phase system kept a stable oxygen concentration over the photocatalyst layer during the reaction.

## 4 SUMMARY AND PERSPECTIVE

In this review, we focused on state‐of‐the‐art approaches in characterizing the interfacial wettability and mass transfer properties in triple‐phase catalysis. Ten types of currently available experimental and computational methods have been summarized. We discussed the advantages, limitations, and applicable scenarios of each method based on their working principle and practical cases. As the emerging of triple‐phase catalysis, this review aims to show the importance and capability of various technologies in evaluating the physicochemical process at catalytic interfaces. However, the characterization still faces several tasks at the current stage.

Optical imaging methods have limited spatial resolution to reveal the fine structure of triple‐phase interfaces at the nanoscale. In contrast, high‐resolution electron microscopy requires a complex preparation process with limited tolerance for low boiling liquid phases. As a result, the experiments can only deliver vague and distorted images, which can hardly reflect the actual structure of a catalytic interface. Besides, the wettability of the catalysts undergoes destruction during catalysis, often leading to significantly changed interfacial structures (e.g., the flooding issue of gas diffusion electrodes).^[^ ^85^ ^]^ The unstable triple‐phase interface often causes poor operational stability, which is one major disadvantage of triple‐phase catalytic systems reported so far. Imaging technologies reported so far have difficulties giving online characterizations on triple‐phase catalysis. Ultrafast cameras have shown the feasibility in revealing the gas bubble adhesion and departure process for gas‐evolving reactions, but cannot provide detailed interfacial structure information due to their relatively low resolution.

For interfacial molecular transfer analysis, most characterizations rely on computational simulations with a large amount of approximate fitting and guesswork. FEM is mainly based on the solution of relevant PDEs according to a series of reaction‐diffusion equilibrium. And empirical correlations are still required in some cases to estimate the interfacial diffusion‐related properties for the simulation. However, the real mass transfer process in triple‐phase catalysis is far more complicated and involves multiple molecule interactions (Van der Waals force, adsorption, chemical bonds, etc.). For example, FEM cannot distinguish boundary conditions sharing the same structure but different wettability and surface energies. Although some experimental methods have been used as compensation to the computational simulations, they can only provide side evidence for the interfacial mass transfer process through detectable signals such as reaction rate, current density, and fluorescence intensity.

Beyond the physical mass transfer process, it is also important to consider the chemical reaction process at triple‐phase interfaces. A deep understanding of the essence of interfacial wettability and its influence on chemical adsorption and activation energies relies on detecting molecule‐catalyst interactions with high temporal and spatial resolutions. The application of environmental transmission electron microscopy and molecular dynamics simulation might be the next breakthrough in characterizing the nature of wettability‐controlled reaction over triple‐phase interfaces at sub‐nano or molecular scale.^[^ ^128^, ^129^, ^130^ ^]^ The integration of two or more characterizations, such as the CLSM‐SEM combination discussed in Section 2.4, shows the opportunity to realize the complementarity between different technologies to provide more powerful experimental evidence. Nonetheless, we firmly believe that constructing theoretical frameworks for the triple‐phase catalysis will be a milestone in the field of catalysis. And developing advanced operando characterization technologies with high temporal and spatial resolutions is the key foundation to fundamentally understand the structure‐controlled interfacial mass transfer and chemical reaction process.

## CONFLICT OF INTEREST

The authors declare no conflict of interest.
